# Bullous Cutaneous Larva Migrans of the Palm

**DOI:** 10.4269/ajtmh.21-1299

**Published:** 2022-03-07

**Authors:** Palaniappan Vijayasankar, Ragavi Subramaniam, Kaliaperumal Karthikeyan

**Affiliations:** Department of Dermatology, Venereology and Leprosy, Sri Manakula Vinayagar Medical College and Hospital, Pondicherry, India

**Figure 1.**
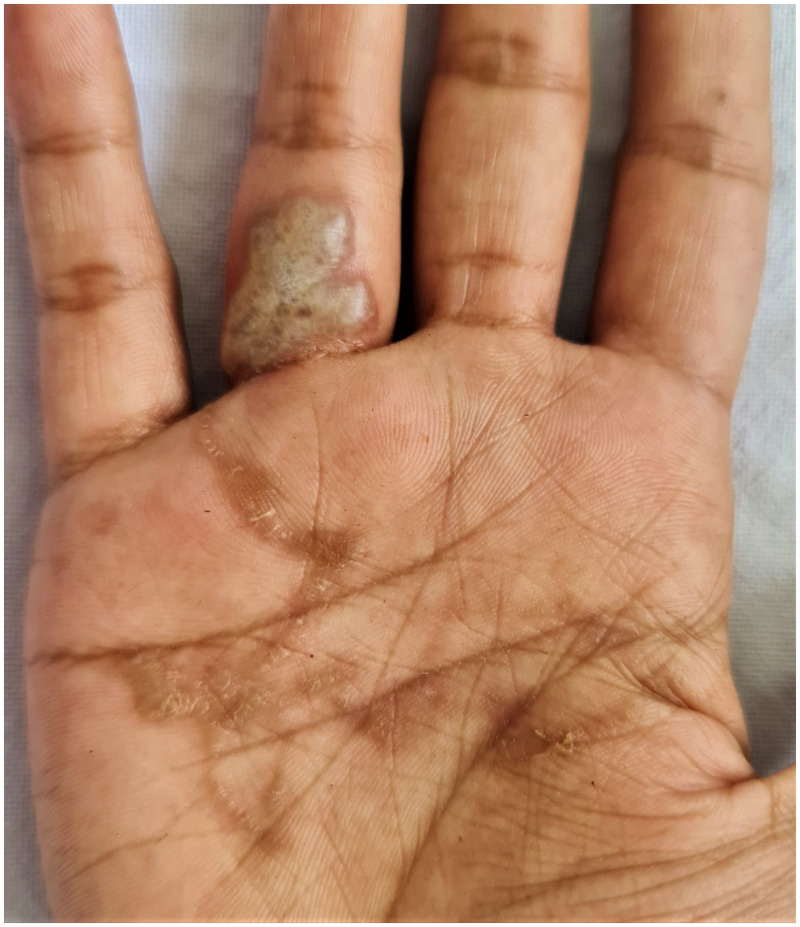
Slightly raised, thread-like, curvilinear tract with a 2 cm × 2 cm bulla over the left palm. This figure appears in color at www.ajtmh.org.

**Figure 2.**
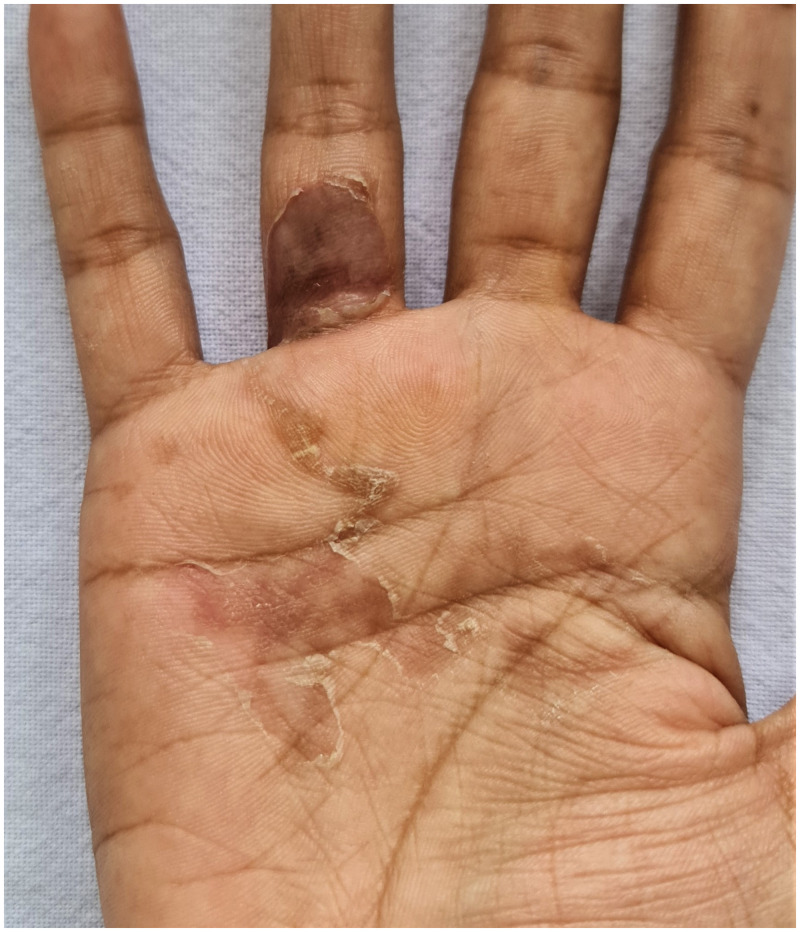
Resolution of bulla and tracts after the treatment. This figure appears in color at www.ajtmh.org.

A 24-year-old woman presented with 1-month history of intensely pruritic creeping eruption over her right palm. Examination revealed a slightly raised, thread-like, curvilinear tract with a 2 cm × 2 cm bulla over the left palm (Figure 1). There was a history of playing in the beach sand 1 month before the onset of eruption. Systemic examination did not reveal any abnormality. Her baseline hematological and biochemical investigations were within normal limits. Stool analysis was negative for parasites. On the basis of the history and clinical picture, a diagnosis of bullous cutaneous larva migrans (CLM) was made. The patient was treated with a single dose of oral ivermectin 12 mg and 3 days albendazole 400 mg daily for 3 days, along with oral antihistamines. The lesions showed resolution in 10 days (Figure 2).

Cutaneous larva migrans is a skin infestation caused secondary to invasion and migration of nematodal larval parasites. This entity is common among tropical and subtropical countries.^1^ The bullous form of CLM is one of the atypical and rare forms with few case reports in literature.^2^^,^^3^ These types of atypical clinical presentations of CLM are currently witnessed more than in the past. The exact pathogenesis of bulla development is not known.^3^ Some authors hypothesized that it might be due to 1) release of lytic enzymes (hyaluronidases and metalloproteases) by the larva, 2) delayed hypersensitivity reaction to unknown antigens released by the larva, and 3) allergic or irritant contact dermatitis to topically applied medicaments.^4^ The cytological examinations of the bulla often reveal lymphocytes, neutrophils, and eosinophils.^3^ Avoiding contact with contaminated soil is the most appropriate preventative measure.^1^ Oral agents such as albendazole, thiabendazole and ivermectin represent the first line of treatment of bullous CLM.^5^
